# Effects of Reorientation of Graphene Platelets (GPLs) on Young’s Modulus of Polymer Nanocomposites under Uni-Axial Stretching

**DOI:** 10.3390/polym9100532

**Published:** 2017-10-20

**Authors:** Chuang Feng, Yu Wang, Sritawat Kitipornchai, Jie Yang

**Affiliations:** 1 School of Engineering, RMIT University, P.O. Box 71, Bundoora, VIC 3083, Australia; chuang.feng@rmit.edu.au (C.F.); s3415279@student.rmit.edu.au (Y.W.); 2 School of Civil Engineering, The University of Queensland, St. Lucia, Brisbane, QLD 4072, Australia; s.kitipornchai@uq.edu.au

**Keywords:** polymer nanocomposites, graphene platelets, uni-axial stretching, micromechanics

## Abstract

The orientation of reinforcement fillers in composites plays a vital role in their mechanical properties. This paper employs the Mori–Tanaka micromechanics model, incorporating the effect of stretching-induced reorientation of graphene platelets (GPL), to predict Young’s modulus of GPL/polymer nanocomposites. Subjected to uni-axial stretching, dispersion of GPLs is described by an orientation distribution function (ODF) in terms of a stretching strain and two Euler angles. The ODF shows that GPLs tend to realign along the stretching direction. Such realignment is enhanced at a higher Poisson’s ratio and under a larger stretching strain. It is found that the out-of-plane Young’s modulus of GPL nanofillers has a limited effect on the overall Young’s modulus of the composites. With an increase in stretching strain and GPL concentration, Young’s modulus increases in the stretching direction while it decreases in the transverse direction. A larger aspect-ratio of GPLs with fewer layers is preferred for enhancing Young’s modulus in the stretching direction, but it is unfavorable in the transverse direction. Moreover, Young’s moduli in both longitudinal and transverse directions are more sensitive to the reorientation of smaller-sized GPLs with a greater concentration in the composites.

## 1. Introduction

Adding graphene and its derivatives into polymers as reinforcements has been observed to dramatically enhance the mechanical properties of the composites and their structures while keeping the advantages of polymers such as flexibility, large deformation, low cost and excellent biological and biochemical compatibility [1,2,3]. This excellent combination promises a novel class of polymer composite materials and structures for various engineering applications, including civil, mechanical, automotive, medical and so on. Extensive studies have been carried out on graphene-based nanocomposites. Rafiee et al. [4] experimentally demonstrated that with a weight fraction (wt) of 0.1%, the Young’s modulus of the graphene-based epoxy nanocomposites can be increased by 31% compared to that of pristine epoxy. Liang et al. [5] found that the tensile strength and Young’s modulus of poly(vinyl alcohol) nanocomposites reinforced by 0.7 wt % of graphene oxide (GO) were increased by 76% and 62%, respectively. The enhancement of strength and toughness of graphene-reinforced epoxy nanocomposites was also experimentally examined by other researchers, including Lee et al. [6], Tang et al. [7], Hu et al. [8] and Yin et al. [9].

Apart from the experimental studies, extensive theoretical work has been done on graphene-based nanocomposites as well. For example, Rahman and Haque [10] developed a molecular-modeling technique to determine the mechanical properties of graphene/epoxy nanocomposites and observed a significant improvement in Young’s and shear moduli of the composites. Ji et al. [11] and Spanos et al. [12] used a micromechanics model to obtain the effective elastic moduli of graphene-reinforced polymer composites. Cho et al. [13] and Shokrieh et al. [14] predicted the mechanical properties of graphene platelet (GPL)/epoxy nanocomposites through a combined molecular dynamic (MD) simulation and micromechanics analysis. In addition to the observation on material properties, the enhancement of graphene and its derivatives on structural behaviours also has been reported [15,16,17,18,19]. The excellent reinforcing effect at a very low content is greatly attributed to the extremely high specific surface areas of graphene and its derivatives, leading to excellent load transfer between the reinforcements and the matrix [4,7,20,21]. Moreover, the mass production and the relatively low cost of graphene’s derivatives, such as GPL and GO, make graphene-based polymer composites promising material candidates in practical engineering applications.

It should be noted that the above-mentioned studies are mainly focused on the manufacturing and characterization of the composites and their structures, with well-dispersed nanofillers, without involving any effects of orientation and distribution state of the reinforcements. However, it has been well-accepted that the orientation and distribution of reinforcing nanofillers have a significant effect on the mechanical properties of the composites [22,23,24,25,26,27,28]. The mechanical properties of the composites can be optimized via manually controlling the realignment of the fillers. In their experimental work, Camponeschi et al. [29] observed improved Young’s modulus of the carbon nanotube (CNT)-reinforced composites when exposed to a magnetic field for realignment. By employing finite element simulation, Joshi et al. [30] found that Young’s modulus of CNT-reinforced nanocomposites is remarkably dependent on the orientation of the dispersed CNTs. The dynamic mechanical analysis by Deniz Ürk et al. [31] demonstrated that the alignment of CNTs increases the longitudinal storage modulus of the composites. The experiments conducted by Li et al. [32] using Raman spectroscopy showed that the Young’s modulus of the nanocomposites with randomly distributed GPLs is lower than that of the nanocomposites where GPLs are perfectly aligned. Several methods, including mechanical stretching [33,34,35], electrical field [36,37,38] and magnetic field [29,30,39], have been well-recognized as effective approaches for the reorientation of nanofillers. It is therefore necessary to investigate the effects of reorientation of the nanofillers on the mechanical properties of the composites. However, research in this area, especially theoretical studies, is quite limited, despite its practical significance.

This paper investigates the effect of uni-axial stretching-induced reorientation of GPLs on Young’s modulus of the nanocomposites through employing a micromechanics model. It is assumed that GPLs are uniformly dispersed without agglomerations before stretching and that the bonding between GPLs and the polymer matrix is perfect. An orientation distribution function (ODF) in terms of a stretching strain and two Euler angles is incorporated into the micromechanics model to capture the influence of reorientation of the nanofillers in the polymer matrix. It should be pointed out that the present work is focused on the influence of reorientation of the nanofillers and the interphase between the nanoparticles and the polymer matrix at nanoscale is not discussed.

## 2. Micromechanics Model

**Figure 1:**
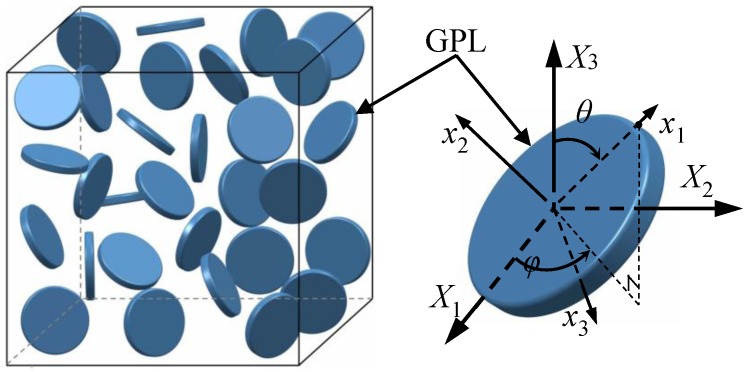
Sketch of a microscale RVE containing GPLs.

For the micromechanics model in this current work, GPLs can be assumed as flat disks with average diameter *d*_GPL_ and thickness *t*_GPL_ [22,40,41]. GPLs are uniformly and randomly dispersed in the polymer matrix with no agglomerations. To determine the effective mechanical properties of the composites with two phases, that is, GPLs and polymer matrix, a representative volume element (RVE) containing enough effective fillers with random orientations, as shown in Figure 1, is selected. This RVE is able to statistically represent the overall properties of the nanocomposites. Based on micromechanics theory, the effective elastic stiffness tensor ***C***_eff_ of the composites can be determined by averaging the terms over all orientations in the RVE [42,43,44] as

(1) $$C_{\mathrm{eff}}=C_{M}+f_{\mathrm{GPL}}\langle \left({\tilde{C}}_{\mathrm{GPL}}-C_{M}\right)\tilde{A}\rangle ,$$

where ***C***_M_ and ***C***_GPL_ are the stiffness tensors of polymer matrix and GPL, respectively, **I** is the identity tensor, *f*_GPL_ is the volume fraction of the GPL, and the angle bracket ‹ › denotes term averaging over all orientations in the global coordinates system of the RVE (i.e., *X*_1_, *X*_2_ and *X*_3_). To simplify the modeling in the present work, the stiffness tensors for GPLs and polymer are reduced to the forms without involvement of tangent stiffness, that is, ***C***_M_ = *E*_M_**I** and ${\tilde{C}}_{\mathrm{GPL}}=\left(E_{\mathrm{in}},E_{\mathrm{out}},E_{\mathrm{in}}\right)I$, where *E*_M_ is Young’s modulus of the polymer matrix and *E*_in_ and *E*_out_ are the in-plane and out-of-plane Young’s moduli of the GPLs, respectively. $\tilde{A}$ is the mechanical concentration tensor in the local coordinate system of the filler (i.e., *x*_1_, *x*_2_, *x*_3_). Using the Mori–Tanaka micromechanics model, the concentration tensor is written as

(2) $$\tilde{A}=\tilde{T}{\left\{\left(1-v_{\mathrm{GPL}}\right)I+f_{\mathrm{GPL}}\tilde{T}\right\}}^{-1},$$

where $\tilde{T}={\left\{I+\tilde{S}{\left({\tilde{C}}_{M}\right)}^{-1}\left({\tilde{C}}_{\mathrm{GPL}}-C_{M}\right)\right\}}^{-1}$ with ***S*** being the Eshelby tensor of GPL. For uncoupled behaviour, this Eshelby tensor is

(3) $$S=\left[\begin{array}{ccc} S_{11} & 0 & 0\\ 0 & S_{22} & 0\\ 0 & 0 & S_{33} \end{array}\right],$$

where $S_{11}=S_{33}=\frac{\pi t_{\mathrm{GPL}}}{4d_{\mathrm{GPL}}}$ and $S_{22}=\frac{\pi t_{\mathrm{GPL}}}{2d_{\mathrm{GPL}}}$, respectively. The average of the concentration tensor over all orientations in Equation (1) can be integrated as

(4) $$\langle \tilde{A}\rangle =\frac{\int_{0}^{2\pi }\int_{0}^{\pi }\tilde{A}\rho \left(\varphi ,\theta \right)\sin\theta d\theta d\varphi }{\int_{0}^{2\pi }\int_{0}^{\pi }\rho \left(\varphi ,\theta \right)\sin\theta d\theta d\varphi },$$

where *φ* and *θ* are polar and azimuth angles, respectively, defining the orientation of GPL in the polymer matrix, and *ρ*(*φ*, *θ*) is the orientation distribution function (ODF) which denotes the probability density of the distribution of GPLs. In particular, *ρ*(*φ*, *θ*) is equal to unity for random distribution, denoting a uniform distribution of GPLs along any orientation.

All quantities in the local coordinate system (*x*_1_, *x*_2_, *x*_3_) can be transformed into the global coordinate system by using a transformation matrix, that is, [45]

(5) $$Q=\left[\begin{array}{ccc} \sin\theta \cos\varphi & -\cos\theta \cos\varphi & \sin\varphi \\ \sin\theta \sin\varphi & -\cos\theta \sin\varphi & -\cos\varphi \\ \cos\theta & \sin\theta & 0 \end{array}\right].$$

Then, the average of the concentration tensor in the global coordinate system can be transformed from its counterpart in the local coordinate system [42,46,47,48] as

(6) $$A=Q^{T}\tilde{T}Q{\left\{\left(1-f_{\mathrm{GPL}}\right)I+\frac{f_{\mathrm{GPL}}}{4\pi }\int_{0}^{2\pi }\int_{0}^{\pi }\left\{Q^{T}\tilde{T}Q\right\}\sin\theta d\theta d\varphi \right\}}^{-1}.$$

Combining Equations (1)–(5), the overall elastic stiffness of GPL/polymer composites can be written as

(7) $$C_{\mathrm{eff}}=C_{M}+\frac{\int_{0}^{2\pi }\int_{0}^{\pi }\rho \left(\varphi ,\theta \right)f_{\mathrm{GPL}}\left(C_{\mathrm{GPL}}-C_{M}\right)A\sin\theta d\theta d\varphi }{\int_{0}^{2\pi }\int_{0}^{\pi }\rho \left(\varphi ,\theta \right)\sin\theta d\theta d\varphi },$$

where $C_{\mathrm{GPL}}=Q^{T}{\tilde{C}}_{\mathrm{GPL}}Q$ is the GPL’s stiffness tensor in the global coordinate system.

## 3. Orientation Distribution Function (ODF)

As mentioned in the Introduction section, spatial orientation of reinforcing nanofillers has significant effects on the mechanical properties of the composites. To quantify the orientation of the fillers, ODF is introduced into the micromechanics model as defined in Equation (7). Particularly, this ODF is unity in case of random and uniform distribution. However, when subjected to mechanical deformation, the ODF, describing the distribution of the reinforcements in the polymer matrix, will no longer be constant but will vary under the applied stretching strain. In what follows, a reorientation model will be adopted to derive the new ODF in terms of the stretching strain and two Euler angles. Regardless of the nanofiller orientation, an ODF is required to satisfy the following conditions [49,50]

(8) $$\rho \left(\varphi ,\theta \right)\geq 0\;\mathrm{and}\;\frac{1}{4\pi }\int_{0}^{2\pi }\int_{0}^{\pi }\rho \left(\varphi ,\theta \right)\sin\theta d\theta d\varphi =1.$$

**Figure 2:**
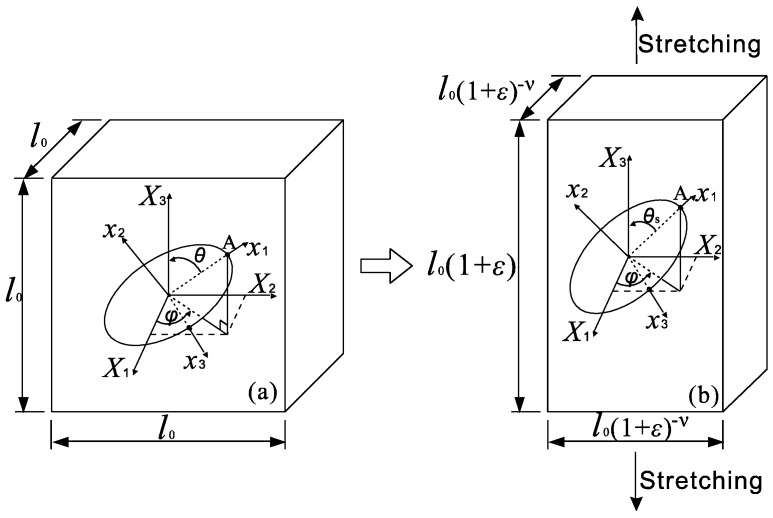
Reorientation of GPL in a cell subjected to uni-axial stretching.

When the composites undergo deformation, the fillers dispersed in polymer matrix will be reoriented due to the load transferred from the matrix. This will result in variations of the filler’s orientation angles, *φ* and *θ*, and the ODF as well. Figure 2 shows a cell containing a GPL filler before and after a uni-axial stretching strain *ε* in *X*_3_ direction. After stretching, the infinitesimal strains in the three dimensions are written as

(9) $$d\varepsilon_{1}=\frac{dx_{1}}{x_{1}},d\varepsilon_{2}=\frac{dx_{2}}{x_{2}}\;,d\varepsilon_{3}=\frac{dx_{3}}{x_{3}}=d\varepsilon .$$

Integrating the three infinitesimal strains over the corresponding elongations, that is, Δ*l*, Δ*w* and Δ*h*, the strains in *X*_1_ and *X*_2_ directions are derived as $\varepsilon_{1}=\varepsilon_{2}={\left(1+\varepsilon \right)}^{-\nu }-1$. Then, the dimensions of the cell become

(10) $$l=l_{0}\left(1+\varepsilon \right),\;w=w_{0}{\left(1+\varepsilon \right)}^{-\nu }\;\mathrm{and}\;h=h_{0}{\left(1+\varepsilon \right)}^{-\nu },$$

where *l*_0_, *w*_0_ and *h*_0_ are the original lengths of the cell before stretching, and *ν* is Poisson’s ratio of the composite. Theoretically, Poisson’s ratio in Equation (10) is dependent on both the orientation and concentration of GPLs. For composites with random distribution of nanofillers, their Poisson’s ratios can be estimated by using the rule of mixture. However, limited work has been done on the determination of Poisson’s ratio in terms of GPL orientation. It is easily understood that Poisson’s ratio of the composite is expected to fall within the range of the values of GPL and the polymer, regardless of the composition of the composites and the orientation of the reinforcements. Therefore, to simplify the modeling, the dependency of Poisson’s ratio on GPL’s concentration and orientation is not considered and three values, that is, 0.1, 0.3 and 0.5, are selected instead to study the influence of Poisson’s ratio on the mechanical properties of the composites.

Due to GPL’s significantly higher stiffness compared to that of the polymer matrix, the deformation of the composites is mainly sustained by the polymer while the composites are subjected to stretching. With GPL’s volumetric expansion being neglected, its volume fraction in the composites is updated as

(11) $$f_{\mathrm{update}}=\frac{V_{0}f_{\mathrm{GPL}}}{V}=\frac{f_{\mathrm{GPL}}}{{\left(1+\varepsilon \right)}^{1-2\nu }},$$

where *V*_0_ and *V* are the volumes of the cell in Figure 2 before and after stretching, respectively. In particular, the volume fraction of GPLs will not change after stretching due to the fact that the composites are incompressible with Poisson’s ratio 0.5.

Under the assumption of perfect bonding without slip between the GPL and the polymer matrix, the GPL inside the cell tends to realign along the stretching direction under the uni-axial stretching, which results in an increase in the polar angle from *θ* to *θ*_s_. However, the variation of the azimuth angle, *φ*, can be neglected in this case [51,52]. The updated polar angle *θ*_s_ can be derived in terms of the initial polar angle *θ* as

(12) $$\theta_{s}=\arctan\left[{\left(1+\varepsilon \right)}^{-\left(1+\nu \right)}\cdot \tan\theta \right].$$

The change of the polar angle after stretching indicates that GPLs tend to realign along the stretching direction, leading to a variation in ODF. For a limiting case, the fillers in the polymer matrix would be perfectly aligned along the stretching direction if the strain is sufficiently large. To determine the new ODF after stretching, it is assumed that there are G nanofillers distributed in the RVE, as shown in Figure 1. The total number of fillers dispersed in the ranges of (*θ*, *θ* + d*θ*) and (*φ*, *φ* + d*φ*) in the RVE can be written [51] as

(13) $$dN_{\begin{array}{l} \theta ,\theta +d\theta \\ \varphi ,\varphi +d\varphi \end{array}}=\frac{1}{4\pi }G\rho \left(\varphi ,\theta \right)\sin\theta d\theta d\varphi .$$

After a stretching, these fillers will be reoriented within the ranges of (*θ*_s_, *θ*_s_ + d*θ*_s_) and (*φ*, *φ* + d*φ*), and the total number [52] is

(14) $$dN_{\begin{array}{l} \theta_{s},\theta_{s}+d\theta_{s}\\ \varphi ,\varphi +d\varphi \end{array}}=\frac{1}{4\pi }G\rho (\varphi ,\theta_{s})\sin\theta_{s}d\theta_{s}d\varphi =dN_{\begin{array}{l} \theta ,\theta +d\theta \\ \varphi ,\varphi +d\varphi \end{array}}.$$

Using Equation (12) and substituting sin*θ*_s_ and d*θ*_s_ into Equation (14), the new ODF *ρ*(*φ*, *θ*_s_) is derived as

(15) $$\rho (\varphi ,\theta_{s})=\frac{{\left(1+\varepsilon \right)}^{\frac{1+\nu }{2}}}{{\left[{\left(1+\varepsilon \right)}^{-\left(1+\nu \right)}\cos^{2}\theta_{s}+{\left(1+\varepsilon \right)}^{1+\nu }\sin^{2}\theta_{s}\right]}^{3/2}}.$$

**Figure 3:**
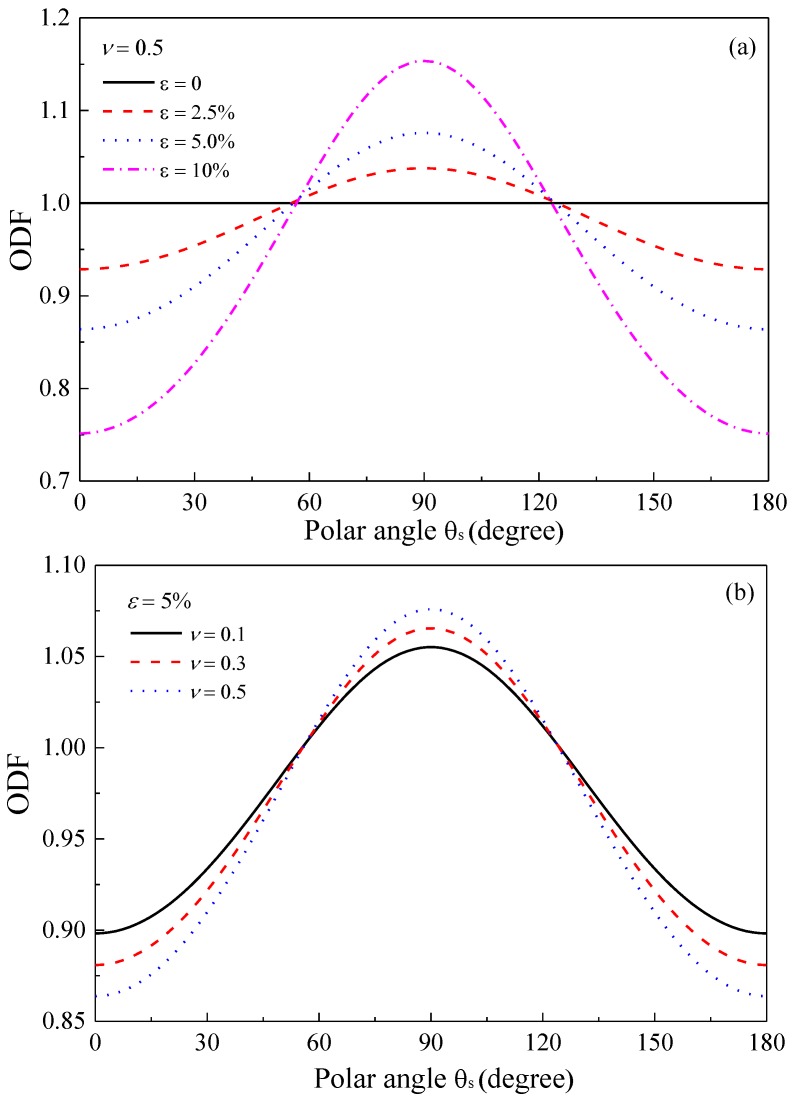
Orientation distribution function (ODF) of GPL (**a**) *ε* = 5%; (**b**) *ν* = 0.5.

As expected, the new ODF *ρ*(*φ*, *θ*_s_) in Equation (15) reduces to unity in the absence of deformation, that is, *ε* = 0. Figure 3 demonstrates the variation in the ODF with polar angle for different Poisson’s ratios and stretching strains. From Figure 3a, it is seen that more GPLs tend to reorient along the stretching direction as the strain increases, which is indicated by the increasing peaks at *θ*_s_ = 90°. Figure 3b investigates the effect of Poisson’s ratio on the ODF. It is found that an increased Poisson’s ratio enhances the realignment of GPLs along the stretching direction. The enhancement of the reorientation behavior can be explained by the dependency of the new polar angle on Poisson’s ratio and the stretching strain, as defined in Equation (12).

## 4. Results and Discussion

**Figure 4:**
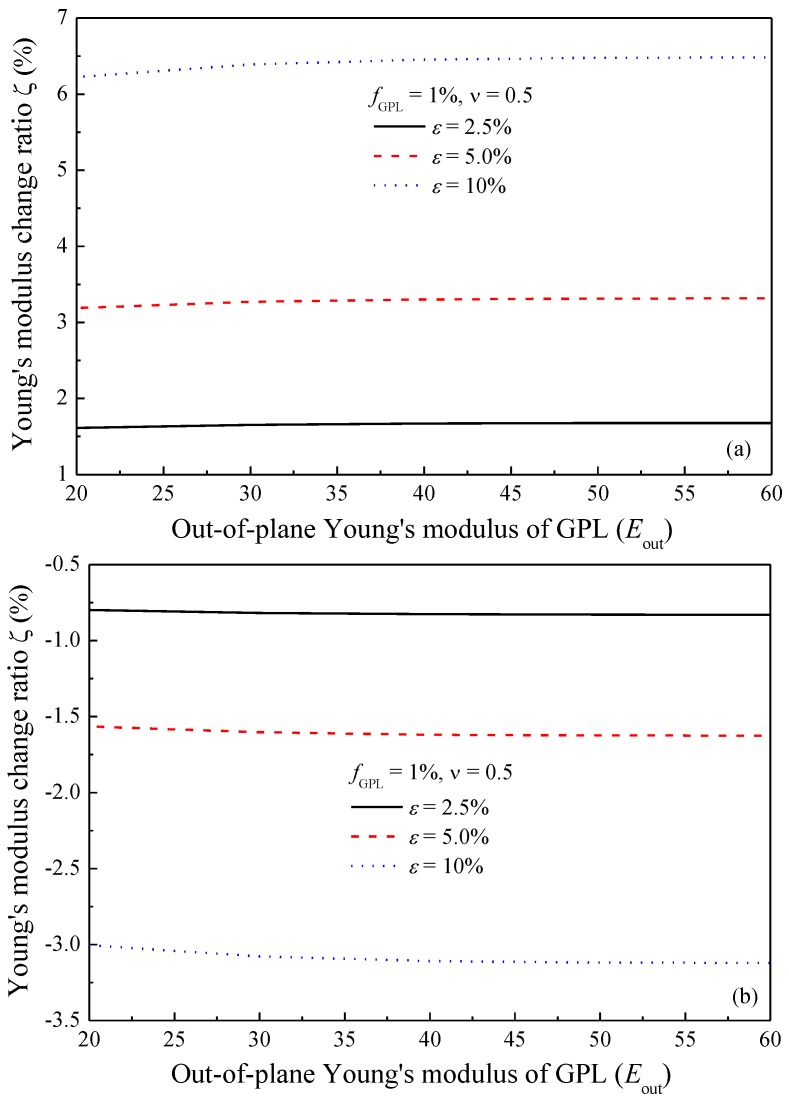
Effect of out-of-plane Young’s modulus of GPL (**a**) stretching direction; (**b**) transverse direction.

The micromechanics model in this current work is first validated. The material properties are chosen as those used by Wang et al. [41] in their experiments. Two grades of GPLs with the same thickness but different diameters, that is, GnP-5 and GnP-C750, are employed to fabricate GPL/epoxy nanocomposites. GnP-5 has an average diameter *d*_GPL_ = 5 μm, thickness *t*_GPL_ = 5–10 nm and surface area of 150 m^2^/g. GnP-C750 has the same thickness as GnP-5 but its diameter is smaller than 1 μm with a surface area of 750 m^2^/g. Epoxy 828 is used as the polymer matrix with *m*-phenylene diamine (*m*-PDA) as the curing agent. After the process of stirring, sonication and milling, the final product demonstrated the appearance of a homogeneous mixture with well-dispersed GPLs. Young’s modulus of the epoxy is *E*_M_ = 2.72 GPa and the in-plane Young’s modulus of GPLs is *E*_in_ = 1.0 TPa. The mass densities of the epoxy and GnP are *ρ*_M_ = 1.2 g/cm^3^ and *ρ*_GPL_ = 2.0 g/cm^3^ [40,41]. GPL’s out-of-plane Young’s modulus *E*_out_ is predicted to be in the range of 20–60 GPa [13], which is argued to be the modulus of exfoliation in the graphite *c*-axis (out-of-plane) [40,53]. Figure 4 shows the variation of Young’s modulus change ratio *ζ* = (*E*_CS_ − *E*_C0_)/*E*_C0_ with the out-of-plane Young’s modulus for stretching and transverse directions. *E*_CS_ and *E*_C0_ denote the overall Young’s moduli of the composites before and after stretching, respectively. It can be seen from this figure that Young’s moduli of the composites in the stretching and transverse directions are not sensitive to the out-of-plane Young’s modulus of GPLs, which agrees with the previously reported observations [11,12,13]. It should be noted that GPL/epoxy composites can only sustain a maximum stretching strain of ~5%. However, the stretching strain for certain polymer matrices, such as Polydimethylsiloxane (PDMS) and rubber, can reach over 100%. For a theoretical study of the orientation effect on Young’s modulus of the composites, a maximum strain of 10% is applied in the present work.

**Figure 5:**
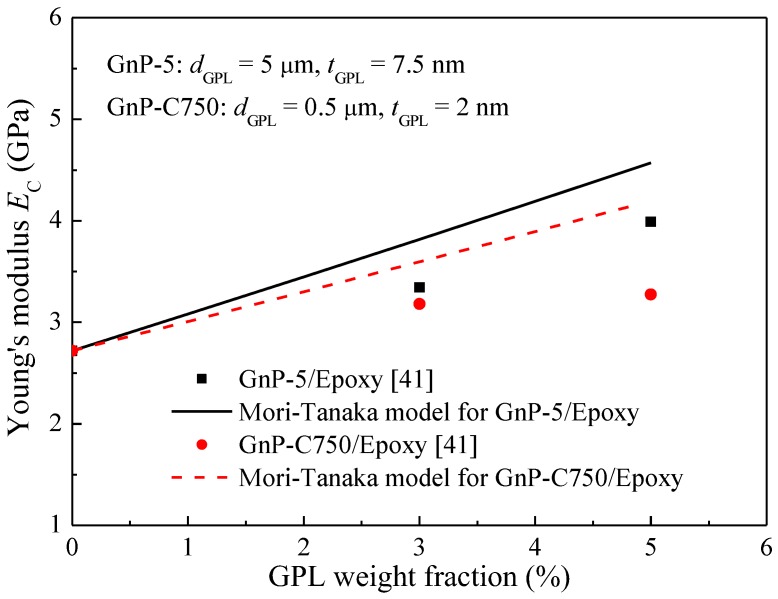
Comparison between present results and experimental data.

To validate the Mori–Tanaka micromechanics model, the present results are compared with the experimental data [41]. The dimensions of the two grades of GPL are set as *d*_GPL_ = 5 μm and *t*_GPL_ = 7.5 nm for GnP-5 and *d*_GPL_ = 0.5 μm and *t*_GPL_ = 2 nm for GnP-C750. It is found from Figure 5 that both experimental and theoretical results indicate that GPLs with a larger aspect ratio have better reinforcing effect on Young’s modulus of the composites compared to their counterparts with a smaller aspect ratio. This phenomenon was explained by Wang et al. [41], that a larger aspect ratio facilitates better load-transfer from the matrix to GPL, thus leading to a higher modulus. Compared with experimental results, the micromechanics model overestimates Young’s modulus for both GnP-5/Epoxy and GnP-C750/Epoxy composites. Such overestimation can be attributed to several reasons. Firstly, it is assumed in the micromechanics model that GPLs are flat disks randomly and uniformly dispersed in the polymer matrix. However, GPLs usually exist in curvature and agglomeration often happens due to their large aspect ratio and the van der Waals force among neighboring GPLs. Both can lead to a reduction in Young’s modulus of the composites. Secondly, the interaction between GPLs and polymer matrix is susceptive to the bubbles, contamination and agglomerations produced during fabrication, which can result in a decreased bonding-strength and load-transfer between the polymer matrix and GPLs. These factors, however, are not taken into account in the model under the assumption of perfect bonding and no slipping.

**Figure 6:**
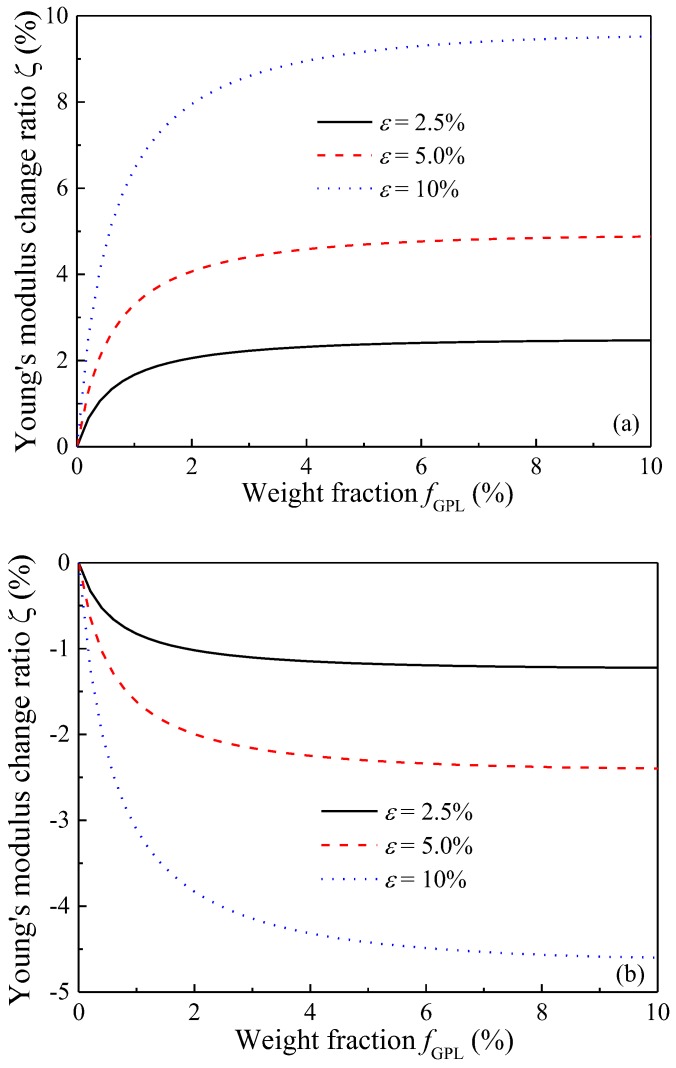
Young’s modulus change ratio of GPL/Epoxy nanocomposites (**a**) stretching direction; (**b**) transverse direction.

Figure 6 plots the variation of Young’s modulus change ratio with GPL weight fraction for the composites under various stretching strains. With an increase in GPL weight fraction, Young’s modulus increases in the stretching direction while it decreases in the transverse direction. This effect becomes more significant when the composites are subjected to a bigger stretching strain. This is because for composites with a higher GPL concentration, the latter tends to realign along the stretching direction. Since GPL’s in-plane Young’s modulus is much higher than its counterpart in the out-of-plane direction, the realignment due to stretching results in an increased Young’s modulus in the stretching direction but a decreased Young’s modulus in the transverse direction. Therefore, the changes of Young’s moduli in the two directions are more sensitive to higher stretching strain for composites with a greater GPL concentration.

**Figure 7:**
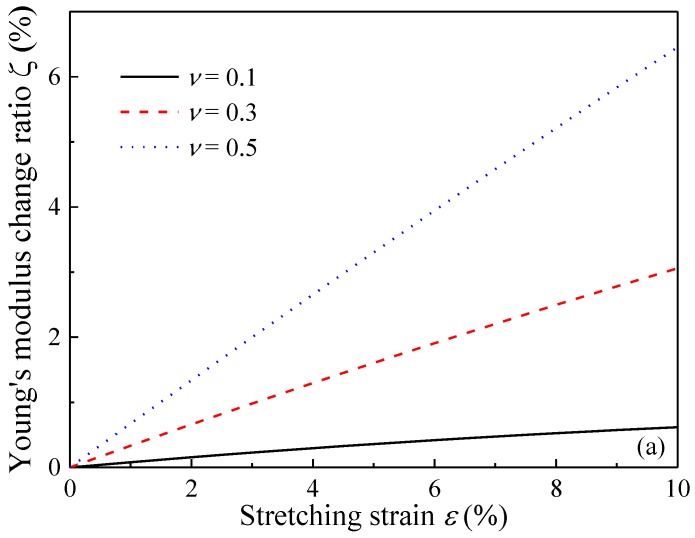

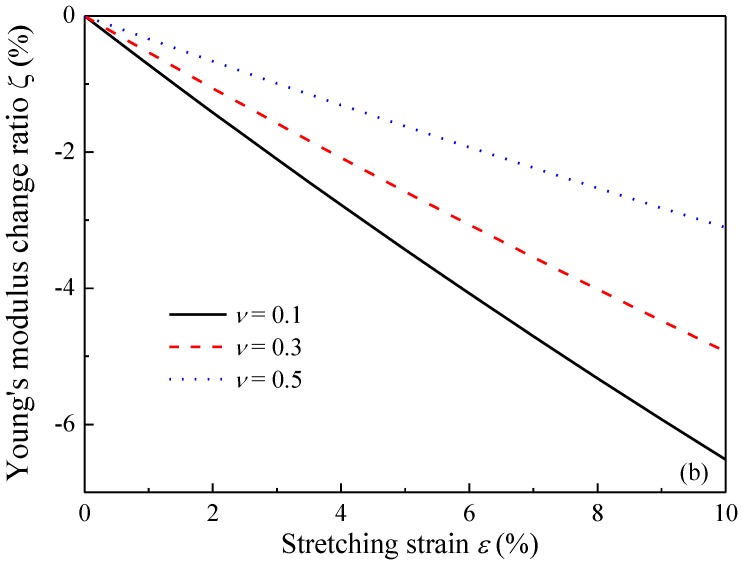
Effect of stretching on Young’s modulus of GPL/Epoxy nanocomposites (**a**) stretching direction; (**b**) transverse direction.

Figure 7 demonstrates that the variation in Young’s modulus in the stretching direction increases, but the value in the transverse direction decreases. In addition, it can be seen that the change of Young’s modulus becomes higher in both of the two directions at a bigger Poisson’s ratio. This phenomenon can be attributed to the dependency of ODF on Poisson’s ratio of the composites as suggested in Figure 3, which indicates that a higher Poisson’s ratio is beneficial for the reorientation of GPL reinforcements.

**Figure 8:**
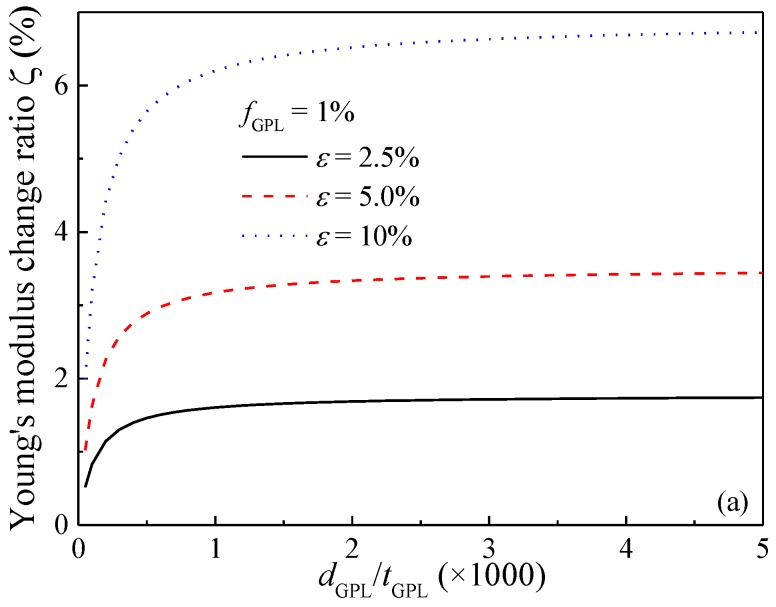

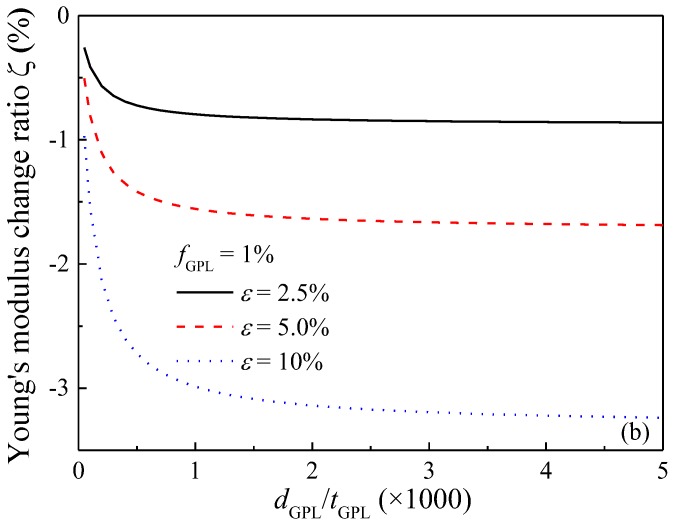
Effect of GPL dimension on Young’s modulus of GPL/Epoxy nanocomposites (**a**) stretching direction; (**b**) transverse direction.

**Figure 9:**
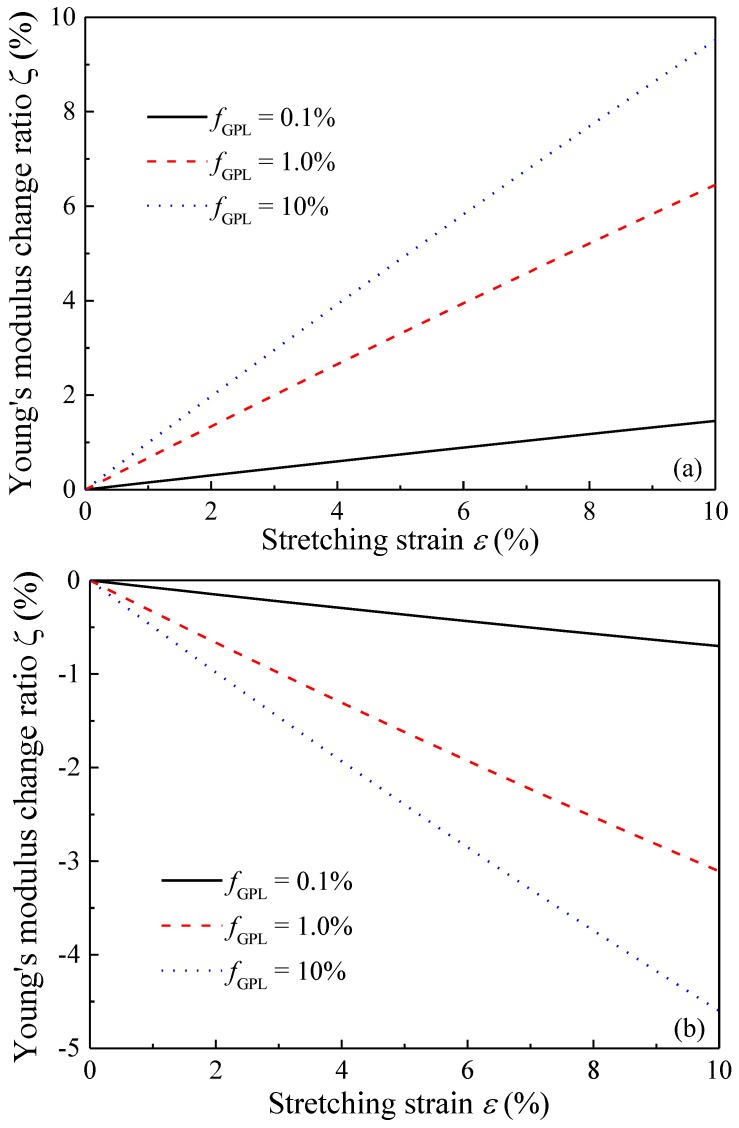
Variation of effective Young’s modulus of GPL/Epoxy nanocomposites with stretching strain (**a**) stretching direction; (**b**) transverse direction.

Figure 8 shows the effect of GPL dimension, in the form of diameter-to-thickness ratio *d*_GPL_/*t*_GPL_, on the Young’s modulus change ratio of the composites subjected to different stretching strains. In this example, the GPL thickness *t*_GPL_ is fixed as a constant while the diameter *d*_GPL_ varies. It is observed that the Young’s modulus increases (decreases) dramatically in the stretching (transverse) directions when the diameter-to-thickness ratio is approximately less than 500, that is, *d*_GPL_/*t*_GPL_ ≤ 500. However, further increase in *d*_GPL_/*t*_GPL_ is seen to have a limited effect. This indicates that Young’s moduli of the composites are more sensitive to the stretching for composites dispersed with smaller-sized GPLs. This is due to the fact that at the same GPL concentration, a smaller size corresponds to more GPL nanofillers dispersed in the matrix, which consequently results in more reorientations when subjected to stretching. The sensitivity of Young’s modulus of the nanocomposites on the GPL weight fraction is investigated in Figure 9. Similar to the trend as previously observed, stretching increases Young’s modulus in the stretching direction while it reduces Young’s modulus in the transverse direction. The change of Young’s modulus in both directions is more sensitive to the stretching for composites with a greater GPL concentration.

## 5. Conclusions

The effects of uni-axial stretching-induced GPL reorientation on Young’s moduli of GPL/polymer composites are investigated by employing the Mori–Tanaka micromechanics model. The orientation distribution of the GPLs in the polymer was characterized by ODF. It was found that GPLs tend to realign along the stretching direction due to the deformation of the RVE when the composites are subjected to stretching. The results show that GPL’s out-of-plane Young’s modulus has a limited effect on the overall Young’s modulus of the nanocomposites, a higher Poisson’s ratio is beneficial for the realignment of GPL nanofillers and such realignment increases Young’s modulus of the composites in the stretching direction while it lowers Young’s modulus in the transverse direction. Given the same stretching strain, larger-sized GPLs are preferred in achieving enhanced Young’s modulus in the stretching direction. Moreover, the variations in Young’s modulus of the composites in both stretching and transverse directions are more sensitive to the stretching when smaller GPLs in a greater concentration are distributed in the matrix.

## References

[1] Kim H., Abdala A.A., Macosko C.W. (2010). Graphene/polymer nanocomposites. Macromolecules.

[2] Papageorgiou D.G., Kinloch I.A., Young R.J. (2015). Graphene/elastomer nanocomposites. Carbon.

[3] Hu K., Kulkarni D.D., Choi I., Tsukruk V.V. (2014). Graphene-polymer nanocomposites for structural and functional applications. Prog. Polym. Sci..

[4] Rafiee M.A., Rafiee J., Wang Z., Song H., Yu Z.-Z., Koratkar N. (2009). Enhanced mechanical properties of nanocomposites at low graphene content. ACS Nano.

[5] Liang J., Huang Y., Zhang L., Wang Y., Ma Y., Guo T., Chen Y. (2009). Molecular-level dispersion of graphene into poly (vinyl alcohol) and effective reinforcement of their nanocomposites. Adv. Funct. Mater..

[6] Lee J.K., Song S., Kim B. (2012). Functionalized graphene sheets-epoxy based nanocomposite for cryotank composite application. Polym. Compos..

[7] Tang L.-C., Wan Y.-J., Yan D., Pei Y.-B., Zhao L., Li Y.-B., Wu L.-B., Jiang J.-X., Lai G.-Q. (2013). The effect of graphene dispersion on the mechanical properties of graphene/epoxy composites. Carbon.

[8] Hu K., Gupta M.K., Kulkarni D.D., Tsukruk V.V. (2013). Ultra-robust graphene oxide-silk fibroin nanocomposite membranes. Adv. Mater..

[9] Yin Y., Hu K., Grant A.M., Zhang Y., Tsukruk V.V. (2015). Biopolymeric nanocomposites with enhanced interphases. Langmuir.

[10] Rahman R., Haque A. (2013). Molecular modeling of crosslinked graphene–epoxy nanocomposites for characterization of elastic constants and interfacial properties. Compos. Part B Eng..

[11] Ji X.-Y., Cao Y.-P., Feng X.-Q. (2010). Micromechanics prediction of the effective elastic moduli of graphene sheet-reinforced polymer nanocomposites. Model. Simul. Mater. Sci. Eng..

[12] Spanos K., Georgantzinos S., Anifantis N. (2015). Mechanical properties of graphene nanocomposites: A multiscale finite element prediction. Compos. Struct..

[13] Cho J., Luo J., Daniel I. (2007). Mechanical characterization of graphite/epoxy nanocomposites by multi-scale analysis. Compos. Sci. Technol..

[14] Shokrieh M., Esmkhani M., Shahverdi H., Vahedi F. (2013). Effect of graphene nanosheets (GNS) and graphite nanoplatelets (gnp) on the mechanical properties of epoxy nanocomposites. Sci. Adv. Mater..

[15] Zhao Z., Feng C., Wang Y., Yang J. (2017). Bending and vibration analysis of functionally graded trapezoidal nanocomposite plates reinforced with graphene nanoplatelets (GPLs). Compos. Struct..

[16] Wang Y., Feng C., Zhao Z., Yang J. (2017). Buckling of graphene platelet reinforced composite cylindrical shell with cutout. Int. J. Struct. Stab. Dyn..

[17] Feng C., Kitipornchai S., Yang J. (2017). Nonlinear bending of polymer nanocomposite beams reinforced with non-uniformly distributed graphene platelets (GPLs). Compos. Part B-Eng..

[18] Feng C., Kitipornchai S., Yang J. (2017). Nonlinear free vibration of functionally graded polymer composite beams reinforced with graphene nanoplatelets (GPLs). Eng. Struct..

[19] Wang Y., Feng C., Zhao Z., Yang J. (2017). Eigenvalue buckling of functionally graded cylindrical shells reinforced with graphene platelets (GPL). Compos. Struct..

[20] Zhao X., Zhang Q., Chen D., Lu P. (2010). Enhanced mechanical properties of graphene-based poly (vinyl alcohol) composites. Macromolecules.

[21] Kuilla T., Bhadra S., Yao D., Kim N.H., Bose S., Lee J.H. (2010). Recent advances in graphene based polymer composites. Prog. Polym. Sci..

[22] Liu H., Brinson L.C. (2008). Reinforcing efficiency of nanoparticles: A simple comparison for polymer nanocomposites. Compos. Sci. Technol..

[23] Krenchel H. (1964). Fibre Reinforcement: Theoretical and Practical Investigations of the Elasticity and Strength of Fibre-Reinforced Materials.

[24] Xie X.-L., Mai Y.-W., Zhou X.-P. (2005). Dispersion and alignment of carbon nanotubes in polymer matrix: A review. Mater. Sci. Eng. R Rep..

[25] Tan S.C., Kwok R.W.O., Chan J.K.W., Loh K.P. (2016). Compression-induced graphite nanoplatelets orientation in fibre-reinforced plastic composites. Compos. Part B Eng..

[26] Kim M., Hwang S.-H., Kim B.-J., Baek J.-B., Shin H.S., Park H.W., Park Y.-B., Bae I.-J., Lee S.-Y. (2014). Modeling, processing, and characterization of exfoliated graphite nanoplatelet-nylon 6 composite fibers. Compos. Part B Eng..

[27] Blanco I., Bottino F.A. (2015). The influence of the nature of posss cage’s periphery on the thermal stability of a series of new bridged poss/ps nanocomposites. Polym. Degrad. Stable.

[28] Blanco I., Bottino F.A., Cicala G., Cozzo G., Latteri A., Recca A. (2015). Synthesis and thermal characterization of new dumbbell shaped poss/ps nanocomposites: Influence of the symmetrical structure of the nanoparticles on the dispersion/aggregation in the polymer matrix. Polym. Compos..

[29] Camponeschi E., Vance R., Al-Haik M., Garmestani H., Tannenbaum R. (2007). Properties of carbon nanotube–polymer composites aligned in a magnetic field. Carbon.

[30] Joshi U.A., Sharma S.C., Harsha S. (2012). Effect of carbon nanotube orientation on the mechanical properties of nanocomposites. Compos. Part B Eng..

[31] Ürk D., Demir E., Bulut O., Çakıroğlu D., Cebeci F.Ç., Öveçoğlu M.L., Cebeci H. (2016). Understanding the polymer type and cnt orientation effect on the dynamic mechanical properties of high volume fraction cnt polymer nanocomposites. Compos. Struct..

[32] Li Z., Young R.J., Wilson N.R., Kinloch I.A., Vallés C., Li Z. (2016). Effect of the orientation of graphene-based nanoplatelets upon the young’s modulus of nanocomposites. Compos. Sci. Technol..

[33] Jin L., Bower C., Zhou O. (1998). Alignment of carbon nanotubes in a polymer matrix by mechanical stretching. Appl. Phys. Lett..

[34] Yao S.-H., Yuan J.-K., Zhou T., Dang Z.-M., Bai J. (2011). Stretch-modulated carbon nanotube alignment in ferroelectric polymer composites: Characterization of the orientation state and its influence on the dielectric properties. J. Phys. Chem. C.

[35] Wang Q., Dai J., Li W., Wei Z., Jiang J. (2008). The effects of cnt alignment on electrical conductivity and mechanical properties of swnt/epoxy nanocomposites. Compos. Sci. Technol..

[36] Wu S., Ladani R.B., Zhang J., Bafekrpour E., Ghorbani K., Mouritz A.P., Kinloch A.J., Wang C.H. (2015). Aligning multilayer graphene flakes with an external electric field to improve multifunctional properties of epoxy nanocomposites. Carbon.

[37] Monti M., Natali M., Torre L., Kenny J.M. (2012). The alignment of single walled carbon nanotubes in an epoxy resin by applying a dc electric field. Carbon.

[38] Ma C., Zhang W., Zhu Y., Ji L., Zhang R., Koratkar N., Liang J. (2008). Alignment and dispersion of functionalized carbon nanotubes in polymer composites induced by an electric field. Carbon.

[39] Wu S., Ladani R.B., Zhang J., Kinloch A.J., Zhao Z., Ma J., Zhang X., Mouritz A.P., Ghorbani K., Wang C.H. (2015). Epoxy nanocomposites containing magnetite-carbon nanofibers aligned using a weak magnetic field. Polymer.

[40] King J.A., Klimek D.R., Miskioglu I., Odegard G.M. (2013). Mechanical properties of graphene nanoplatelet/epoxy composites. J. Appl. Polym. Sci..

[41] Wang F., Drzal L.T., Qin Y., Huang Z. (2015). Mechanical properties and thermal conductivity of graphene nanoplatelet/epoxy composites. J. Mater. Sci..

[42] Taya M. (2005). Electronic Composites: Modeling, Characterization, Processing, and Mems Applications.

[43] Odegard G., Gates T., Wise K., Park C., Siochi E. (2003). Constitutive modeling of nanotube–reinforced polymer composites. Compos. Sci. Technol..

[44] Benveniste Y. (1987). A new approach to the application of mori-tanaka’s theory in composite materials. Mech. Mater..

[45] Entchev P.B., Lagoudas D.C. (2002). Modeling porous shape memory alloys using micromechanical averaging techniques. Mech. Mater..

[46] Feng C., Jiang L. (2013). Micromechanics modeling of the electrical conductivity of carbon nanotube (cnt)–polymer nanocomposites. Compos. Part A Appl. Sci. Manuf..

[47] Seidel G.D., Lagoudas D.C. (2009). A micromechanics model for the electrical conductivity of nanotube-polymer nanocomposites. J. Compos. Mater..

[48] Feng C., Jiang L. (2015). Micromechanics modeling of bi-axial stretching effects on the electrical conductivity of cnt-polymer composites. Int. J. Appl. Mech..

[49] Pérez R., Banda S., Ounaies Z. (2008). Determination of the orientation distribution function in aligned single wall nanotube polymer nanocomposites by polarized raman spectroscopy. J. Appl. Phys..

[50] van Gurp M. (1995). The use of rotation matrices in the mathematical description of molecular orientations in polymers. Colloid Polym. Sci..

[51] Kuhn W., Grün F. (1942). Beziehungen zwischen elastischen konstanten und dehnungsdoppelbrechung hochelastischer stoffe. Kolloid-Zeitschrift.

[52] Feng C., Jiang L.Y. (2014). Investigation of uniaxial stretching effects on the electrical conductivity of cnt–polymer nanocomposites. J. Phys. D Appl. Phys..

[53] Marsh H., Rodriguez-Reinoso F. (2001). Sciences of Carbon Materials.

